# The difference of regulatory effect of two *Inonotus obliquus* extracts on high‐fat diet mice in relation to the fatty acid elongation function of gut microbiota

**DOI:** 10.1002/fsn3.2012

**Published:** 2020-11-24

**Authors:** Jian Yu, Hongyu Xiang, Qiuhong Xie

**Affiliations:** ^1^ Key Laboratory for Molecular Enzymology and Engineering of Ministry of Education School of Life Sciences Jilin University Changchun Jilin China; ^2^ National Engineering Laboratory for AIDS Vaccine School of Life Sciences Jilin University Changchun Jilin China; ^3^ School of Life Sciences Jilin University Changchun Jilin China

**Keywords:** fatty acid elongation, fatty acid metabolism, *Inonotus obliquus*, obesity

## Abstract

Obesity is a disease that causes metabolic disorders in the human body and is closely related to intestinal microbes. This experiment compares the therapeutic effects of two *Inonotus obliquus* extracts on high‐fat diet (HFD) mice and explores the effects and mechanisms of intestinal flora and its metabolites. The energy intake (EI), weight gain (BWG), fecal flora diversity, fecal and urine metabolites, and fecal triglycerides (TG) of mice were measured at 4 temporal points. We found that due to the difference in energy intake between the two groups in the early stage of the experiment, the ethanol extract of *Inonotus obliquus* (IOE) had a stronger effect on the accumulated BWG than the polysaccharide (IOP) of *Inonotus obliquus* at the end of the experiment. Moreover, the difference caused by IOE and IOP intake was the largest in the second week, in four temporal points. Compared with IOP, IOE in the second week can reduce EI, fecal short‐chain fatty acids (SCFA) and TG, reduce host metabolism, increase fecal *Akkermansia* and fatty acid elongation, and increase host substrate phosphorylation. The change trend of the fatty acid elongation P value from 2 to 14 weeks is consistent with the overall difference trend between the two groups. The difference in the regulating effect of the two *Inonotus obliquus* extracts on HFD mice is related to the fatty acid elongation function of the intestinal flora, which leads to the reduction of IOE and the effect of BWG is better than IOP. It provides a theoretical reference for the development of functional food using the extract of *Inonotus obliquus*.

## Graphical abstract

The fatty acid elongation function of bacteria (especially Akkermansia) increased MCFA and decreased SCFA. Increasing MCFA reduced energy intake and weight gain. Reducing SCFA reduced the level of oxidative phosphorylation, TCA cycle, and nutrient metabolism and increased the level of substrate phosphorylation.

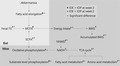

## 1 INTRODUCTION

Obesity is a metabolic disease characterized by metabolic disorder of host and gut microbiota (Canfora et al., 2019a, 2019b). It is caused by a complex interaction of environmental factors such as genes, diet, food composition, and lifestyle (Fall & Ingelsson, 2014; Gerard, 2016). With the development of science and technology and the abundance of materials, a large amount of high energy density food had entered people's lives, resulting in a large number of obese individuals. One of the World Health Organization (WHO) surveys showed that more than 18% of children (380 million) and 39% of adults (1.9 billion) were overweight or obese ((WHO), 2016). Obesity can cause type 2 diabetes, gout, Alzheimer's disease (Iyengar et al., 2016; Ng et al., 2014; Pedditzi et al., 2016), and many other diseases, and it was related to over 60% of deaths(Reilly, 2017). In recent years, many studies have found that plant and mushroom extracts improved obesity safely and effectively (Ganesan & Xu, 2018; Martel et al., 2017).

Gut microbiota mainly lives in the mammalian large intestine, and they are closely related to the health of the host(Gerard, 2016). Moreover, studies have shown that plant and mushroom extracts improve obesity through gut microbiota (Chang et al., 2015; Gao et al., 2018; Jiao et al., 2019). In the high‐fat environment, the extracts affect the intestinal flora, including lipid sensitive microorganisms (such as *Akkermansia*, *Escherichia coli*), lipid metabolism (such as fatty acid metabolism, bile acid metabolism), and related metabolites (such as short‐chain fatty acids, medium chain fatty acids) (Kitazume et al., 2008; H. Zhang et al., 2006). In addition, previous studies have shown that different extracts have different effects on obese individuals and their gut microbiota (Lu et al., 2017). In order to further understand the weight loss mechanism of the extracts, it is necessary to compare the effects of different extracts on the metabolism of host and gut microbiota.

*Inonotus obliquus* is a mushroom for both medicine and food, and it has been made into a healthy tea drink by the Russians for centuries. (Duru et al., 2019b). The extracts from *Inonotus obliquus* have good effect on metabolic diseases (Duru et al., 2019a; Han et al., 2019). *Inonotus obliquus* ethanol extract (IOE) is rich in polyphenols and triterpenes, and the I. obliquus polysaccharides (IOP) are rich in polysaccharides. The two extracts had different effects on obese individuals due to different active ingredients (Yu et al., 2020). In this paper, we compare the effects of the two extracts on HFD mice at four temporal points (weeks 0, 2, 8, and 14) to explore more potential mechanisms.

## 2 MATERIALS AND METHODS

### 2.1 Preparation of IOE/IOP

IOP (containing 78.27% polysaccharide) was obtained by hot water extraction, 80% ethanol precipitation, protein removal, and ethanol cleaning, and IOE (containing 27.86% polyphenols and 38.55% triterpenoids) was extracted by 80% ethanol after removing impurities with hot water (Yu et al., 2020).

### 2.2 Animal experimental design

The experimental protocol was approved by the Animal Ethics Committee of Jilin University and complied with national laws. Five‐week‐old C57BL/6J male mice (15–17 g; Beijing Vital River Laboratory Animal Technology Co., Ltd., China) were housed in a controlled environment (24 ± 1°C, 12‐hr daylight cycle) and fed with food and water randomly. After 10 days of acclimation, the mice were fasted overnight (12 hr) for the determination of the fasting blood glucose. Subsequently, the mice were divided into four groups (NCD group, HFD group, the IOE group, and the IOP group) according to their body weights and fasting glucose levels. And the mice were kept in their original cages as much as possible because changing residences may result in aggressive behaviors. The mice in NCD group were fed with normal chow diet, and the mice in HFD group, the IOE group, and the IOP group were fed with high‐fat diet. The mice in the IOE group were fed by gavage with IOE at a dose of 500 mg/kg per day, and the mice in the IOP group were by gavage with IOP at a dose of 1,000 mg/kg per day. The dosage of IOP refers to previous studies, the dosage of IOE refers to the dosage of IOP, and the ratio of the extraction rate of the two extracts (6.25%: 3.09%). The body weight and food intake were assessed weekly. After 14 weeks of treatment, the mice were sacrificed for specimens. Specimens were stored at −80°C for future use.

### 2.3 Sample collection

Urine and feces were collected using metabolic cages at weeks 0, 2, 8, and 14, and 50 μL sodium azide solution (0.1% w/w) was added into each urine sample. All samples were then stored in a −80℃ freezer for later analysis.

All samples were thawed at room temperature. For each urine sample, 400 μL of the sample was mixed with a solution of 200 μL PBS in H_2_O. 500 μL of supernatants was pipetted into NMR analysis tubes after centrifuging (15,000 rpm, 5 min, 4°C). 50 μL D_2_O containing TSP was also added to each tube. The fecal samples were homogenized with 1 ml of PBS (containing TSP) per 0.1–0.2 gram of stool. 600 μL of supernatants was freeze‐dried after centrifuging (15,000 rpm, 15 min, 4°C) twice. Each powder was pipetted into NMR analysis tubes after mixing with 600 μL D_2_O and passing through 0.22‐μm membrane filters. D_2_O provided a field frequency lock and TSP a chemical shift reference (1H, δ 0.0).

### 2.4 Fecal TG

The fecal TG was measured by using kits obtained from the Nanjing Jiancheng Bioengineering Institute (Nanjing, China).

### 2.5 NMR data acquisition and processing

All samples were analyzed with AVANCE III 600M MHz NMR spectrometer at 298.2 K. 1H NMR spectra were acquired by the 1D version CPMG (fecal samples) and noesyphpr (urine samples) pulse sequence with water suppression during the relaxation delay of 3 s and a mixing time of 0.1 s. Sixty‐four free induction decays were collected into 64 K data points with a spectral width of 7,812.5 Hz (fecal samples) and 8,417.5 Hz (urine samples), with an acquisition time of 2 s. FID was zero‐filled to 64 K prior to Fourier transformation.

All the spectra were manually phased and baseline corrected in software MestreNova 12.0 (Mestre‐lab Research SL). Each spectrum was segmented into regions with a width of 0.005 ppm between δ 9.6 and δ 0.4. The δ 5.48–6.20 region in urine spectra and the δ 4.72–5.20 region in all spectra were excluded to eliminate the effects of urea signals and water suppression. All remaining regions of the spectra were then normalized to the total sum of the integrated spectral area to reduce any significant concentration differences.

### 2.6 Sequencing and function prediction of fecal microbiota

DNA extraction, sequencing, and data processing were performed by using a previously described method (Y. Wang et al., 2018).

We used PICRUSt (Phylogenetic investigation of communities by reconstruction of unobserved states) to perform functional predictions. PICRUSt generates metagenomic predictions from 16S rRNA data using annotations of sequenced genomes in the IMG database. And the Kyoto Encyclopedia of Genes and Genomes (KEGG) database was used for functional classification.

### 2.7 Data reuse

Some of the figures in this paper and the author's already‐published papers were based on the same data (Yu et al., 2020), including body weight, EI, and urine data at week 14.

### 2.8 Statistical analysis

The data were expressed as means ± standard errors of the means (*SEM*). The P value between two independent groups was analyzed by using an unpaired two‐tailed *t* test. The histogram was drawn by using GraphPad Prism 8 (GraphPad Software, La Jolla, CA). The STAMP software was used to compare the gut microbiota. Metabolomics data were subjected to OPLS‐DA by using the software SIMCA 14.0 (Umetrics, Sweden) to construct multivariate statistical models.

### 2.9 Accession number

High‐throughput sequencing data have been submitted to the NCBI Sequence Read Archive (SRA) under the accession number PRJNA576716.

## 3 RESULTS

### 3.1 The effect of IOE/IOP on BWG and EI at week 14

**Figure 1:**
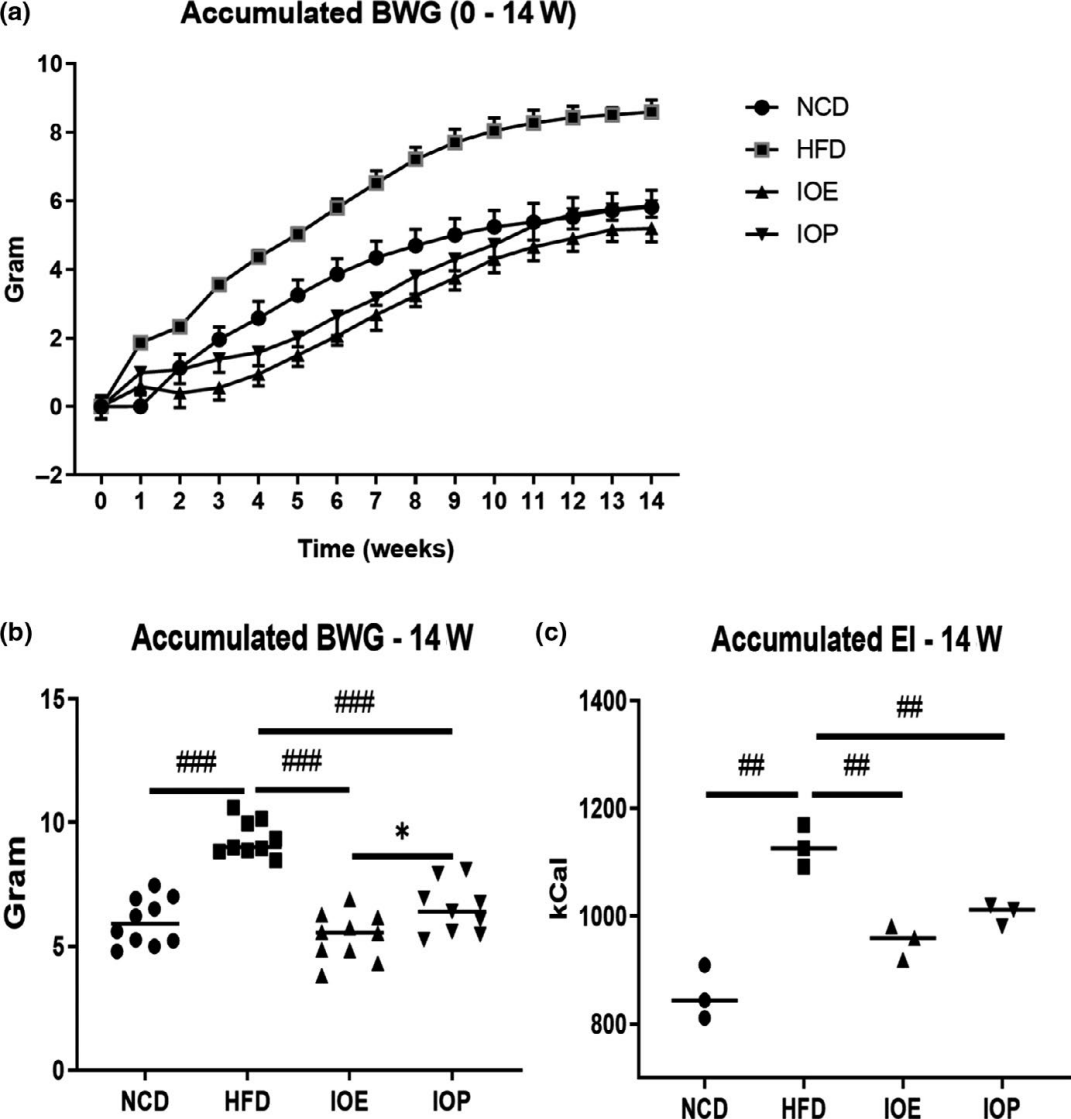
Effects of IOE/IOP administration on BWG and EI of the HFD mice. (A) BWG curves (0 ‐ 14w); (B) Accumulated BWG in 14 weeks; (C) Accumulated EI in 14 weeks. #:*p* < .05 (versus. HFD); ##:*p* < .01 (versus. HFD); ###:*p* < .001 (versus. HFD); *:*p* < .05 (IOE versus. IOP); **:*p* < .01 (IOE versus. IOP); ***:*p* < .001 (IOE versus. IOP)

During the 14 weeks of animal experiments, the mice in HFD group showed higher weight growth rate than the mice in NCD group (Figure 1a). At the end of the experiment, the BWG and EI of the HFD mice were more than those of the NCD mice (Figure 1b–c). Both *Inonotus obliquus* extracts significantly improved the body weight growth rate, accumulated BWG, and accumulated EI of HFD mice within 14 weeks. Moreover, we found that the inhibitory effect of IOE on accumulated BWG (14 weeks) in HFD mice is significantly stronger than IOP, while there was no obvious difference in their effect on EI.

### 3.2 The effect of IOE/IOP on mice and gut microbiota at week 14

Previous studies have shown that the regulation effect of mushroom active substances on host metabolism is related to the gut microbiota and its metabolites. Therefore, we sequenced the 16s rRNA of the fecal microbiota and determined the metabolites in feces and urine using ^1^H NMR‐based metabolomics.

**Figure 2:**
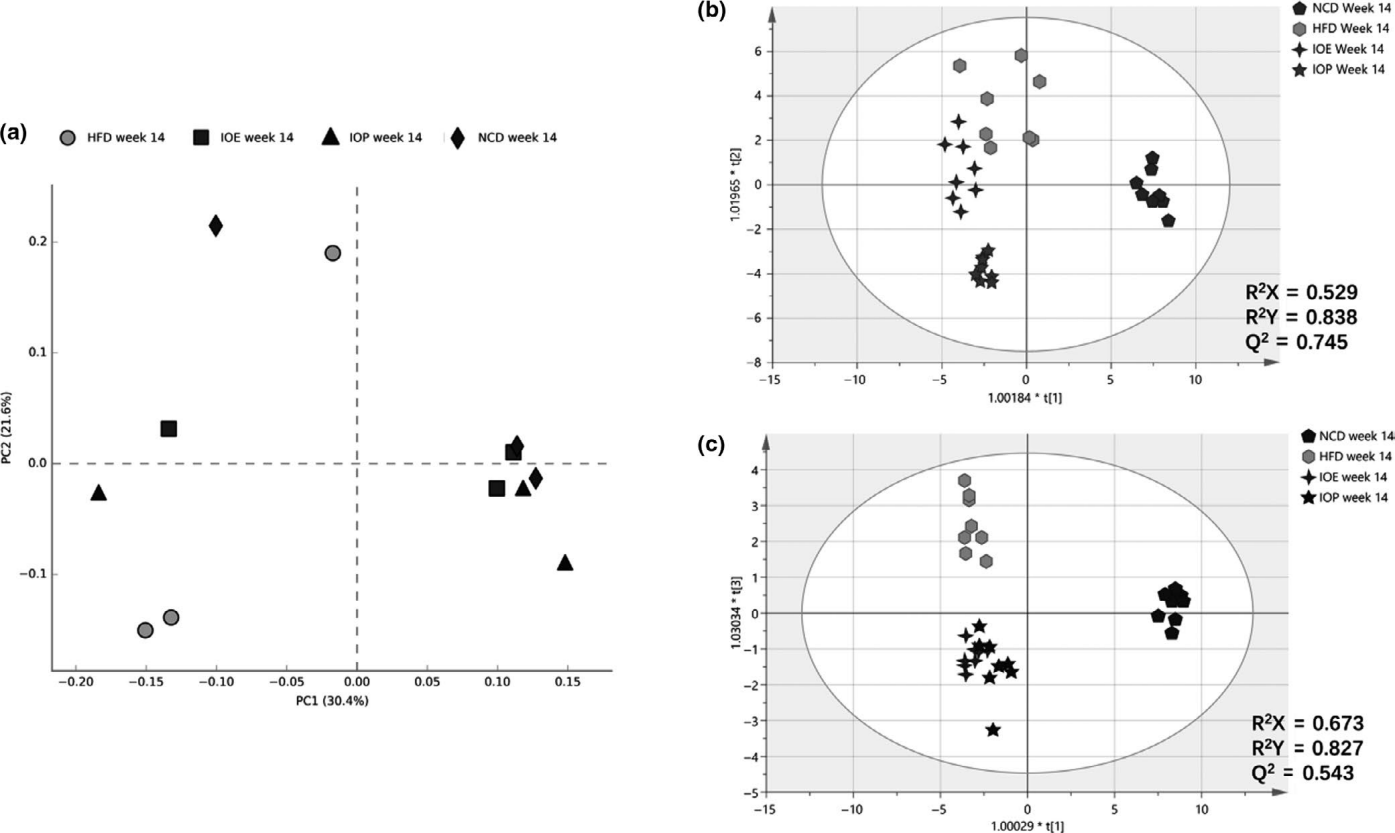
Comparison of each group under different conditions. (A) Score scatter plots of the PCA classification of fecal microbiota at week 14; (B) score scatter plots of the PCA classification of fecal microbiota in the IOE group and the IOP group; (C) score scatter plots of the OPLS‐DA classification of fecal metabolites at week 14

The PCA score plot was used to describe the fecal microbial difference in the four groups at the 14th week. The fecal samples of the HFD group not only did not separate significantly from the samples of the NCD group, but also did not separate well from the samples of the IOE group or the IOP group (Figure 2a). We selected gut microbes with an average relative abundance greater than 1% for analysis (Figure S1–S2). At the phylum level, Firmicutes in the IOE group was significantly more than that in the HFD group, while Firmicutes and Actinobacteria in the IOE group were significantly more than those in the IOP group. However, no statistical difference (HFD versus NCD/IOE/IOP, IOE versus IOP) was found in the comparison of 17 gut microbial genera with an average relative abundance greater than 1% (Figure S2). Moreover, no significant difference (HFD versus NCD/IOE/IOP, IOE versus IOP) was found in the comparison of various predictive functions (class 2) of the metabolism (class 1) of the gut microbiota (Figure S3).

The metabolomics data of feces and urine were analyzed by OPLS‐DA. The fecal samples of the four experimental groups were well separated, including the HFD group and the other three groups, the IOE group and the IOP group (Figure 2b). In the hydrogen spectrum, we distinguished 23 fecal metabolites (Figure S4), and we selected the metabolites with VIP greater than 2 for statistical analysis (Figure S4). Both IOE and IOP intake significantly changed lactate, glucose, and choline in the feces of HFD mice. The branched amino acids in the feces of HFD mice were also changed due to IOE intake, while the intake of IOP caused significant changes in butyrate, lysine, 5‐aminovaleric acid, and bile acids in the feces of HFD mice. Moreover, the fecal metabolites that were significantly different between the IOE group and the IOP group were lysine, 5‐aminovalerate, glucose, and bile acids (Figure S5).

In the scatter plot of urine samples, the samples of the IOE group or IOP group were significantly separated from the HFD group, but the samples of the two groups were not well separated (Figure 2c). We identified 27 urine metabolites, of which 16 metabolites had VIPs greater than 2. Both IOE and IOP intake changed the cis‐aconitic acid, pyruvate, arginine,, and 3‐ureidopropionate in the urine of HFD mice, and there was a significant difference in pyruvate between the two groups.

The above results indicate that the intestinal flora and urine metabolite group of IOE group and IOP group were not much different, while the fecal metabolite groups of the two groups are significantly different.

### 3.3 Comparison of IOE group and iop group at several temporal points

In the data analysis of intestinal flora and urine metabolites at week 14, we found that the method of comparing the data of the two groups with the HFD group may not be as good as the direct comparison between the data of the two groups. In order to further investigate the difference between the effects of IOE and IOP on HFD mice and gut microbiota, we chose to collect data again for analysis at four temporal points including week 0 (the beginning of the experiment), week 2 (the early stage of the experiment), and week 8 (the middle of the experiment).

**Figure 3:**
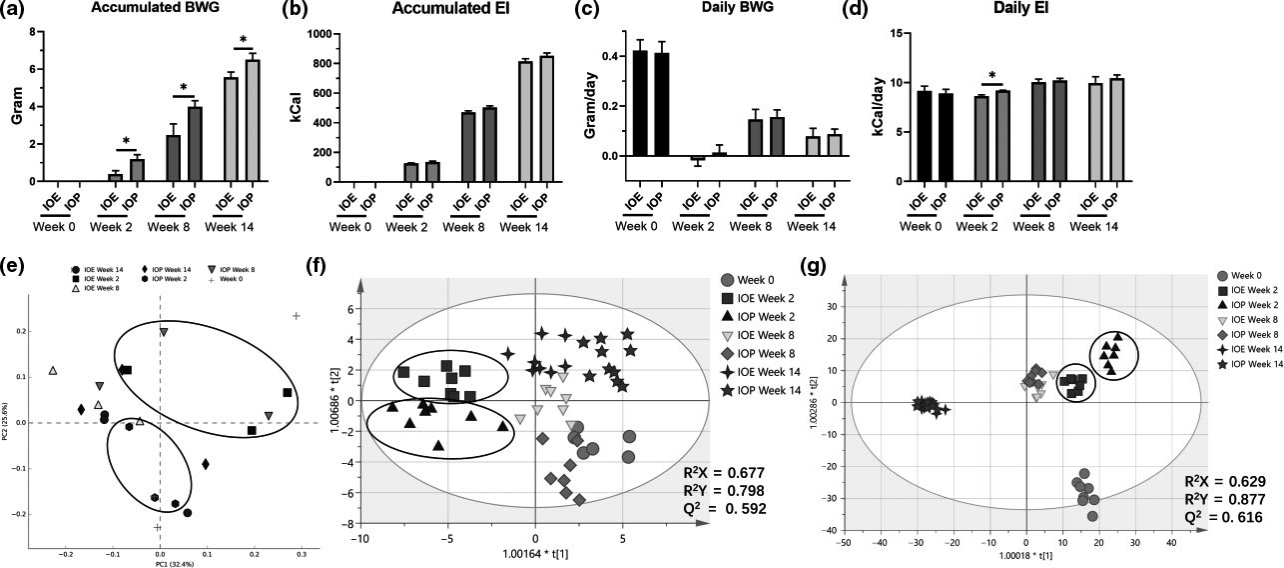
(A) Accumulated BWG of mice in the IOE group and the IOP group in 0, 2, 8, and 14 weeks; (B) Accumulated EI of the IOE group and the IOP group in 0, 2, 8, and 14 week; (C) BWG of the IOE group and the IOP group at week 0, 2, 8, and 14; (D) EI of the IOE group and the IOP group at week 0, 2, 8, and 14; (E) score scatter plots of the OPLS‐DA classification of fecal metabolites in the IOE group and the IOP group; (F) score scatter plots of the OPLS‐DA classification of urine metabolites at week 14; (G) score scatter plots of the OPLS‐DA classification of urine metabolites in the IOE group and the IOP group

We found that the accumulated BWG of the IOE group was significantly lower than that in the IOP group in 2 weeks (*p* = .0146), 8 weeks (*p* = .0398), and 14 weeks (*p* = .0486) after the beginning of the experiment (Figure 3a), while there was no significant difference in accumulated EI between the two groups at four temporal points (Figure 3b). Then, the daily changes of at the four temporal points were studied. We did not find any significant difference in daily BWG between the two groups at week 2, at week 8, and at week 14 (Figure 3c). However, the daily EI of the IOE group was significantly less than that in the IOP group at week 2, while there was no significant difference in daily EI between the two groups at week 8 and at week 14 (Figure 3d).

In the comparison of intestinal microbes between the IOE group and the IOP group, we found that the samples of the two experimental groups in the second week were significantly separated. Although the samples of the IOE group and the IOP group at week 8 were distinguished, the difference between the two groups was not as obvious as the week 2. As in the previous paragraph, we found that the fecal microbial samples of the IOE group and the IOP group were not significantly different in this comparison (Figure 3e).

In the score scatter plot used to analyze fecal metabolites, the samples of the IOE group and the IOP group were significantly different at week 2, 8, and 14 (Figure 3f).

After analyzing the urine metabolite samples by OPLS‐DA, we found that the samples of IOE group and IOP group were well separated at the second week, while the samples of the two groups were partially overlapped at the eighth week. Moreover, the analysis results at week 14 were consistent with those in the previous paragraph.

These results show that the biggest difference between the IOE group and the IOP group occurred at week 2, and the smallest difference occurred at week 14.

### 3.4 Analysis of gut microbiota and its functions at week 2

**Figure 4:**
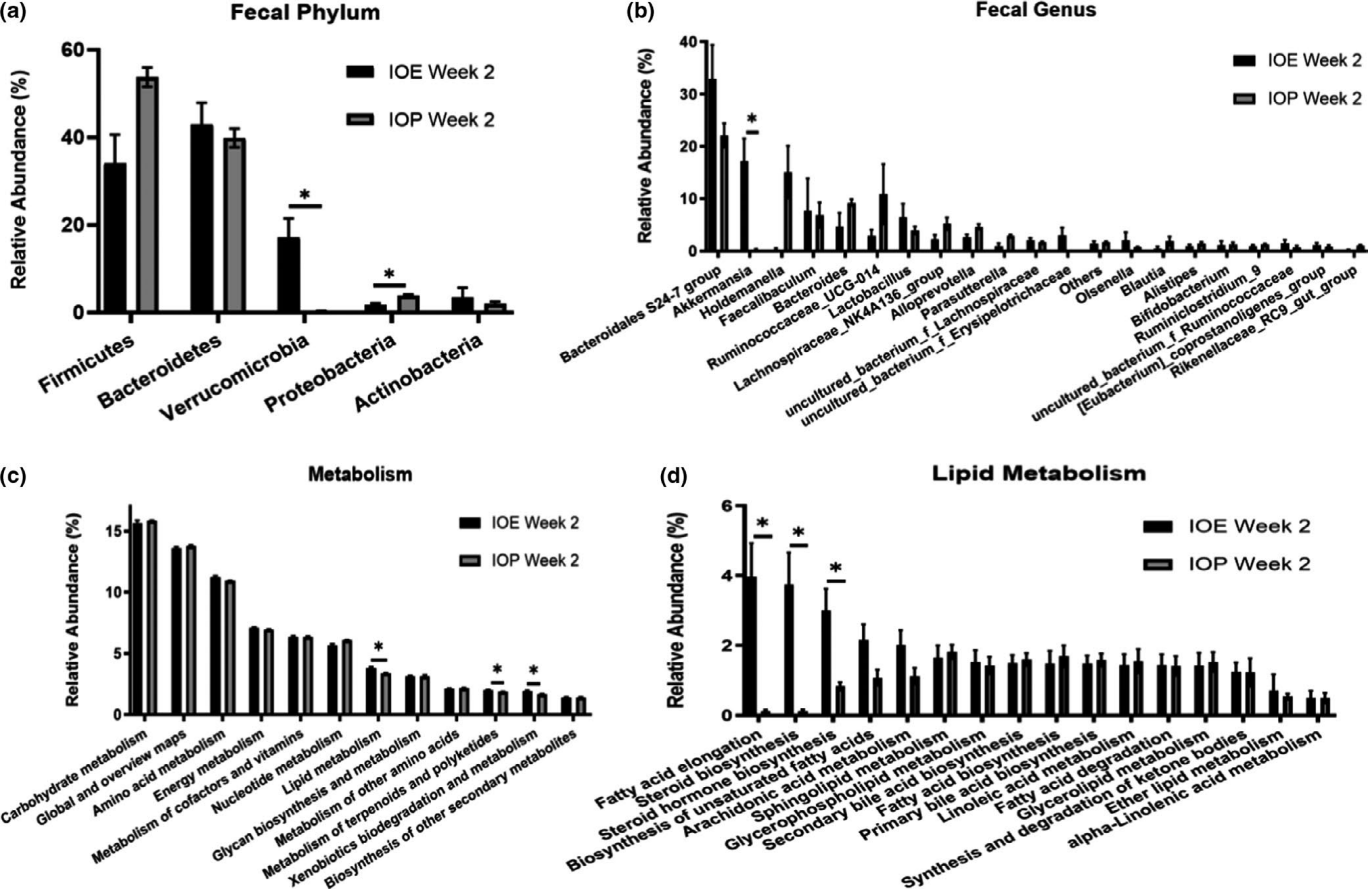
Analysis of gut microbiota and its functions at week 2. (A) Statistical analysis of microbiota at the phylum level; (B) Statistical analysis of microbiota at genus level (D); (E) Statistical analysis of predictive function (Class 2) of metabolism (Class 1); (F) Statistical analysis of predictive function (Class 3) of lipid metabolism (Class 2). *:*p* < .05 (IOE versus. IOP); **:*p* < .01 (IOE versus. IOP); ***:*p* < .001 (IOE versus. IOP)

The microbial composition (relative abundance > 1%) was shown in Figure S8a‐b. At the phylum level, the microbiota of the five groups was dominated by species of the phyla *Firmicutes*. *Bacteroidetes*, *Proteobacteria*, *Verrucomicrobia,* and *Actinobacteria* (Figure S8a). Compared with the IOP group, the IOE group had higher *Verrucomicrobia* and lower *Proteobacteria* in feces (Figure 4a). At the genus level, there were 16 species of microbiota with relative abundance more than 1%, including *Bacteroidales* S24–7 group, *Akkermansia*, *Holdemanella*, *Faecalibaculum*, and *Bacteroides* (Figure S8b). Moreover, the relative abundance of *Akkermansia* in the IOE group was significantly higher than that in the IOP group (Figure 4b).

We used PICRUSt to predict the functional composition of a microbial community's metagenome based on the 16S rRNA profile at week 2. Three different predicted gene functions (Class 2) of metabolism (Class 1) in the IOE group were significantly higher than those in the IOP group. The three prediction functions include Lipid metabolism, Metabolism of terpenoids and polyketides, and Xenobiotics biodegradation and metabolism (Figure 3e). Moreover, compared with the IOP group, the more abundant predicted gene functions (Class 3) of lipid metabolism (Class 2) in the IOE group included fatty acid elongation, steroid biosynthesis, and steroid hormone biosynthesis (Figure 3f).

The results indicated that the difference of structure and function of fecal bacteria caused by intake of IOE and IOP was manifested in *Akkermansia* and lipid metabolism.

### 3.5 Metabolic analysis of hfd mice and gut microbiota at week 2

The fecal metabolites were divided into metabolites related to SCFA, glucose metabolism, lipid metabolism, and amino acid metabolism. Among SCFA related metabolites, propionate and butyrate in the IOE group were significantly less than those in the IOP group, while there was no significant difference in acetate and methanol (SCFA metabolites) between the two groups. Compared with the IOP group, the IOE group had more lysine and 5‐aminovalerate (degradation products of lysine), less glutamate, and branched amino acids and glycine without significant difference. Moreover, there was no significant difference in the five metabolites related to glucose metabolism (lactate, succinate, and glucose) and lipid metabolism (bile acids and choline) between the two groups (Figure 4c). However, after determination of TG in feces, we found that TG in the IOE group were less than those in the IOP group (Figure 4d). These results suggest that, compared with IOP, IOE intake reduces fecal SCFA and changes fecal amino acid.

**Figure 5:**
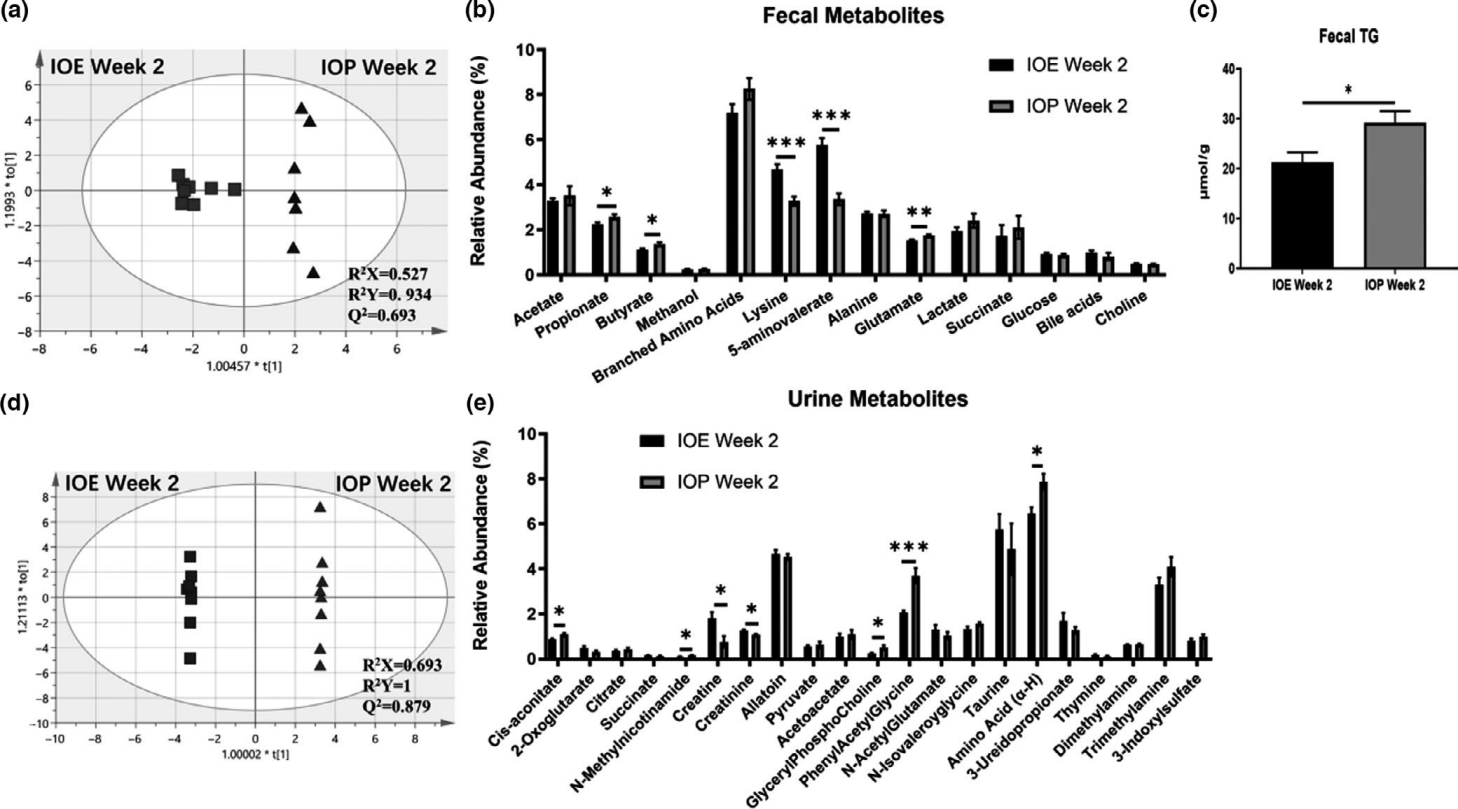
Metabolic analysis of gut microbiota and mice in the IOE group and the IOP group at week 2. (A) Score scatter plots of the OPLS‐DA classification of fecal samples; (B) Statistical analysis of main metabolites (VIP > 2) identified through^1^H NMR data of fecal samples; (C) Fecal TG (D) Score scatter plots of the OPLS‐DA classification of urine samples; (E) Statistical analysis of main metabolites (VIP > 2) identified through^1^H NMR data of urine samples. *:*p* < .05 (IOE versus. IOP); **:*p* < .01 (IOE versus. IOP); ***:*p* < .001 (IOE versus. IOP)

Compared with the IOP group, the urine content of cis‐aconitate (TCA cycle), N‐Methylnicotinamide (synthetic substances for NADH), Glyceryl Phospho Choline (lipid metabolism), Phenyl Acetyl Glycine (by‐products of fatty acid degradation), and amino acids (Amino acid metabolism) in the urine of the IOE group were more abundant, and there were more creatine and creatinine (substrate level phosphorylation) in the IOE group (Figure 5c). The results indicated that the mice in the IOE group had lower the production of NADH, TCA cycle and metabolism of the three major nutrients levels, and higher substrate phosphorylation levels.

### 3.6 Change of p value

To explore the change trend of the difference between the IOE group and the IOP group, we calculated the P values of twelve key data at several temporal points (Table 1). The P value of accumulated BWG and butyrate has been less than 0.05 at three temporal points. From week 2 to week 8, the p value of 7 indexes changed from less than 0.05 to more than 0.05. Among them, the P value of fatty acid elongation changed from 0.0167 to 0.0521 (*p* < .1). From week 8 to week 14, the difference in *Akkermansia* and creatinine between the two groups changed from significant (*p* < .05) to insignificant (*p* > .05). And, the P value of fatty acid elongation in week 14 was 0.3318.

**Table 1:** P value of key data

Key data	*p* Values at week 2	*p* Values at week 8	*p* Values at week 14
Accumulated BWG	**0.0146**	**0.0398**	**0.0486**
EI	**0.0187**	0.7024	0.5319
Akkermansia	**0.0333**	**0.0405**	0.3463
Fatty acid elongation	**0.0167**	0.0521	0.3318
Propionate	**0.0035**	0.3896	0.7316
Butyrate	**0.0337**	**0.0226**	**0.0311**
TG	**0.0195**	0.5651	0.1107
Cis‐aconitate	**0.0372**	0.6886	0.9121
NNMD	**0.0421**	0.1096	0.1275
Creatinine	**0.0301**	**0.0342**	0.3609
Amino acid (α‐H)	**0.0095**	0.6030	0.3169
PAG	**0.0005**	0.5096	0.7172

The change of P value reflected the trend of difference between IOE group and IOP group.

## 4 DISCUSSION

In week 14 of this experiment, we found that both IOE and IOP reduced the BWG of HFD mice, and IOE has a stronger effect on BWG of HFD mice than IOP. Using the method of comparing the administration group (IOE group and IOP group) and the positive control group (HFD group), IOE and IOP showed no significant difference in intestinal flora (genus level) and urine metabolites. Therefore, the data at week 14 are difficult to form a chain of evidence to explain why IOE is more effective in reducing BWG in HFD mice than IOP.

Throughout the experiment (week 1 to week 14), the effects of these two extracts on the BWG of HFD mice had always been different, and this difference was getting smaller with time. Moreover, the EI of the IOE group was less than that of the IOP group in the second week. These results indicate that the difference in accumulated BWG of the IOE group and the IOP group started from the difference in daily EI between the two groups in the second week. In addition, we found that the difference in the effects of the two extracts on the HFD mice and gut microbiota was the largest at week 2. Furthermore, the difference between the impact of IOE and IOP is gradually getting smaller from week 2 to week 8 and then to week 14. Therefore, we started from the second week of data analysis, in order to explore the reasons for the difference between the BWG of the IOE group and the IOP group.

After summarizing all the statistically different data in the second week of this experiment, we found that most of the data of the microbiota were related to fatty acid metabolism, because high‐fat diet contained a lot of TG rich in long‐chain fatty acids. In addition, the data related to fatty acid metabolism include *Akkermansia*, fatty acid elongation, SCFA, and TG.

*Akkermansia* is the only genus of *Verrucomicrobia (T. Zhang, Li, Cheng, Buch, & Zhang, 2019)* and easily affected by the lipid content of the diet. Many studies have revealed the mechanism by which *Akkermansia* affects obese individuals. These mechanisms are related to SCFA (Zhai et al., 2018), gut permeability (Chelakkot et al., 2018), chronic inflammation (Boulangé et al., 2016), etc. In addition, *Akkermansia* is often described as a mucous membrane‐degrading bacterium (Derrien et al., 2017), but few people have mentioned the fatty acid elongation genes that are abundant in the genome of *Akkermansia* (https://www.kegg.jp/kegg‐bin/show_pathway?amu01100). It is still unknown whether *Akkermansia* affects obese individuals is related to its fatty acid elongation gene. Surprisingly, *Akkermansia* and fatty acid elongation was highlighted in the data comparison between the IOE group and the IOP group at week 2 of this experiment.

Fatty acid elongation is a series of genes related to carbon chain elongation of fatty acids. The genes cause different results in different environments (Zarins‐Tutt et al., 2016). In the lipid‐rich intestine, with the increase of fatty acid elongation and the decrease of SCFA, the enrichment of SCFA appeared at the same time (Wu et al., 2018). Moreover, we also found that fecal TG (rich in long‐chain fatty acids) and SCFA in the IOE group were less than those in the IOP group. These results also suggested that the medium chain fatty acids (MCFA) of the IOE group were higher than that of the IOP group. In addition, it is proven that MCFA will inhibit the host's EI, because MCFA promotes the secretion of intestinal anorexia hormones (glucagon‐like peptide and peptide YY) through free fatty acid receptors of intestinal L cells (Hara et al., 2014). Although SCFA can also promote the secretion of intestinal hormones through free fatty acid receptors (Bohan et al., 2019; Chambers et al., 2015), the binding ability of MCFA to the receptor is greater than that of SCFA (Hara et al., 2014). The above results and analysis explain why the EI of the IOE group was less than that of the IOP group at week 2.

SCFAs, the main metabolite of gut microbiota, were significantly different between healthy individuals and obese individuals (Ahmadi et al., 2019; S. Wang et al., 2017). They can bring many metabolic benefits to obese individuals, perhaps because SCFA can promote oxidative phosphorylation of the host (Rose et al., 2018). Oxidative phosphorylation, the most important energy metabolism pathway in host, produces ATP from coenzyme NADH and FADH_2_. The coenzymes are mainly produced in TCA cycle, the common pathway of three nutrients metabolism. In addition, if the ATP produced by oxidative phosphorylation pathway is insufficient, the body will promote other metabolic pathways to supplement ATP, such as substrate level phosphorylation. In the second week of the experiment, the SCFA level of the IOE group was significantly lower than that of the IOP group. Moreover, the production of NADH, TCA cycle, and metabolism of the three major nutrients also showed lower levels in the IOIE group. Correspondingly, the phosphorylation level of the IOE group was higher than that of the IOP group. These results are consistent with previous studies.

**Figure 6:**
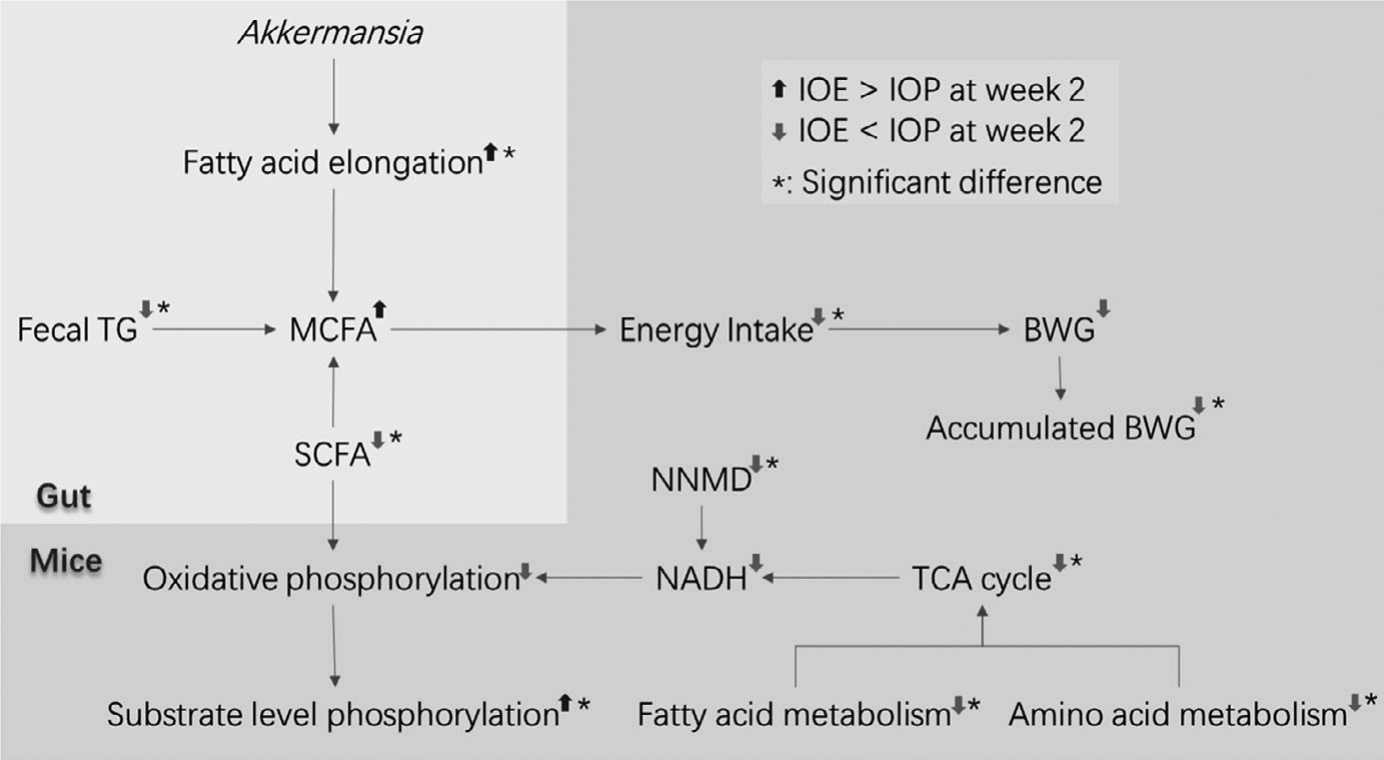
Schematic overview of the difference between IOE and IOP at week 2

In summary (Figure 6), *Akkermansia* affected the metabolism of the HFD mice through fatty acid elongation based on the comparison of the data of the IOE group and the IOP group at week 2. The increase in *Akkermansia* leads to an increase in the fatty acid elongation gene, thereby increasing the production of MCFA. And MCFA reduced the BWG of the HFD mice by suppressing EI. At the same time, the increase of MCFA was closely related to the decrease of fecal TG and SCFA. And then, the host's oxidative phosphorylation also dropped due to SCFA. Following this, the host metabolic level decreased (the production of NADH, TCA cycle, and metabolism of the three major nutrients) and the ATP production pathway level increased (substrate level phosphorylation).

Finally, we found that the change process of the fatty acid elongation difference (P value) between the IOE group and the IOP group is consistent with the change process of the overall difference between the two groups. Combined with the discussion about the data association in the second week, we believed that the fatty acid elongation function of the flora was the key data for the difference between IOE and IOP throughout the experiment.

## 5 CONCLUSION

In contrast, IOE increased the EI of HFD mice through *Akkermansia* and its fatty acid elongation gene in the second week, while IOP enhanced the energy metabolism of HFD mice through SCFA. As a result, IOE was better than IOP in reducing BWG in HFD mice. The difference in the regulatory effects of the two *Inonotus obliquus* extracts on HFD mice was related to the fatty acid extension function of the intestinal flora. The correlation provides a theoretical reference for how to use *Inonotus obliquus* extract to develop functional foods.

## CONFLICTS OF INTEREST

The sponsors are not involved in any of the design, execution, interpretation, or writing of the study.

## AUTHOR CONTRIBUTIONS

J.Y., H.X., and Q.X. conceptualized the study. J.Y. investigated the study. H.X. and Q.X. involved in supervision and resources. J.Y. involved in writing–original draft.

## Supporting information

Figures S1–S8

## Data Availability

Some or all data, models, or code generated or used during the study are available from the corresponding author by request.
