# Supplementary material for: The difference of regulatory effect of two Inonotus obliquus extracts on high‐fat diet mice in relation to the fatty acid elongation function of gut microbiota
Source: Food Sci Nutr. 2020 Nov 24;9(1):449–58. doi: 10.1002/fsn3.2012 (PMC7802550; doi:10.1002/fsn3.2012)
Supplement: Supplementary file 1 — Figures S1–S8 [file FSN3-9-449-s001.pdf]

Supplemental Figure

THE DIFFERENCE OF REGULATORY EFFECT OF TWO INONOTUS OBLIQUUS EXTRACTS ON HIGH FAT-DIET MICE IN RELATION TO THE FATTY ACID ELONGATION FUNCTION OF GUT MICROBIOTA

Jian Yu [3], Jun-Yan Xiang [3], Hongyu Xiang [1,2,3*], Qiuhong Xie [1,2,3*]

[1] Key Laboratory for Molecular Enzymology and Engineering of Ministry of Education, School of Life Sciences, Jilin University, Changchun, Jilin 130012, People's Republic of China

[2] National Engineering Laboratory for AIDS Vaccine, School of Life Sciences, Jilin University, Changchun, Jilin 130012, People's Republic of China

[3] School of Life Sciences, Jilin University, Changchun, Jilin 130012, People's Republic of China

* Corresponding authors:

Hongyu Xiang, Professor; E-mail: hyxiang@jlu.edu.cn

Qiuhong Xie, Professor; E-mail: qhxie@jlu.edu.cn

Tel & Fax: +86-431-85153832

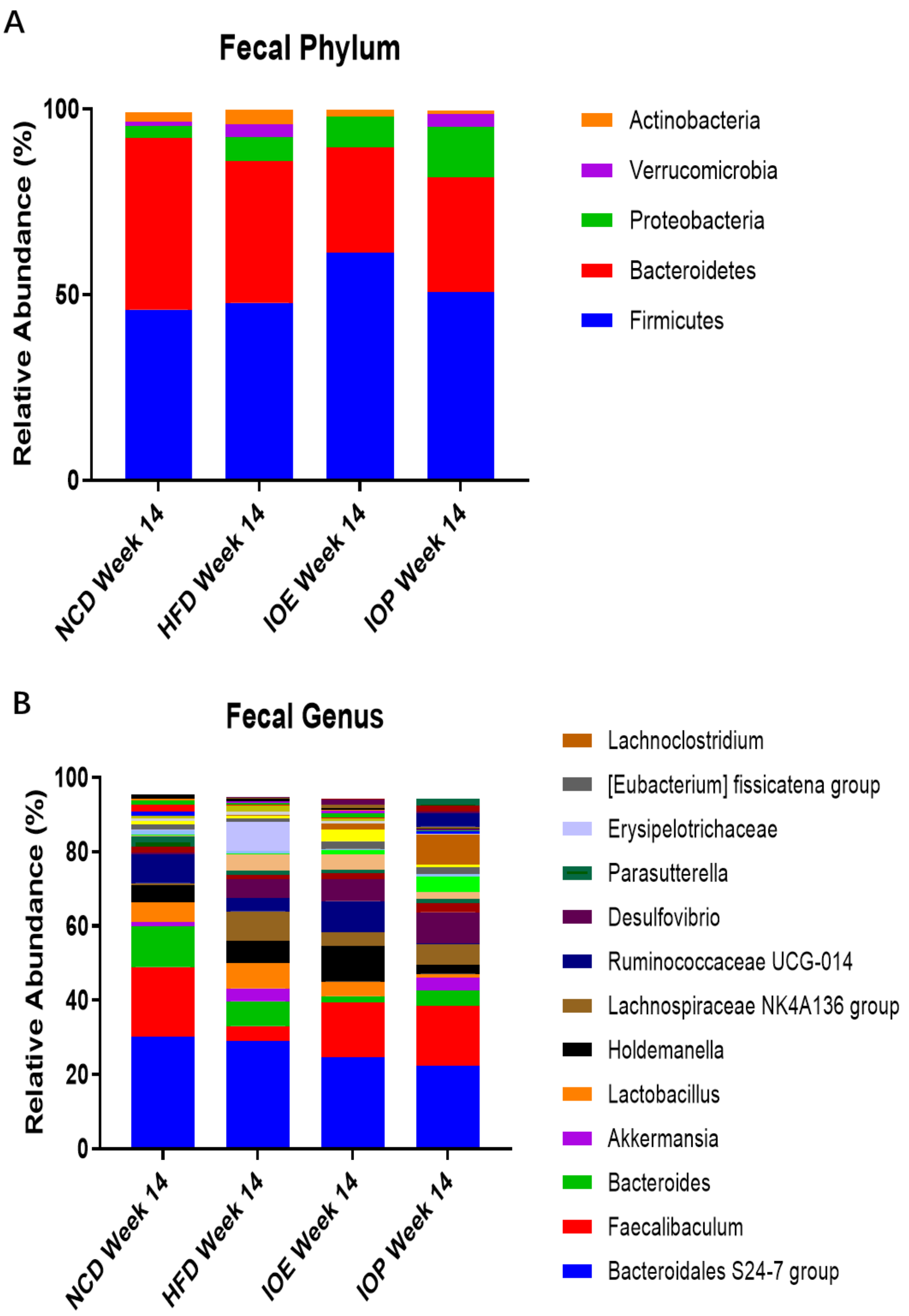

Figure S1. The composition of gut microbiota at week 14. (A) The composition of gut microbiota at phylum level; (B) The composition of gut microbiota at genus level

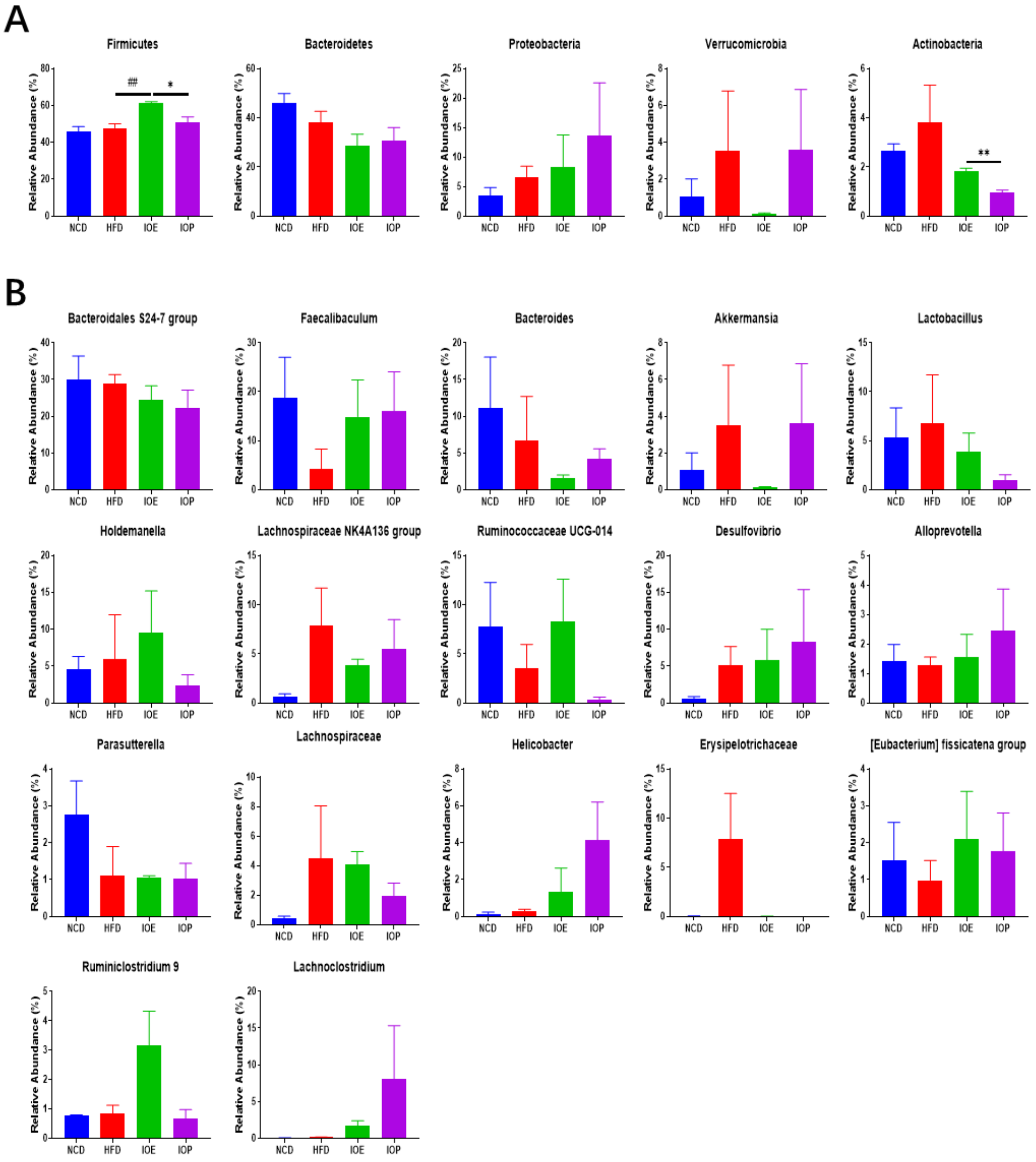

Figure S2. Statistical analysis of gut microbial relative abundance at week 14. (A) Statistical analysis of microbiota at the phylum level; (B) Statistical analysis of microbiota at the genus level. #: P<0.05 (vs. HFD); ##: P<0.01 (vs. HFD); ###: P<0.001 (vs. HFD); *: P<0.05 (IOE vs. IOP); **: P<0.01 (IOE vs. IOP); ***: P<0.001 (IOE vs. IOP).

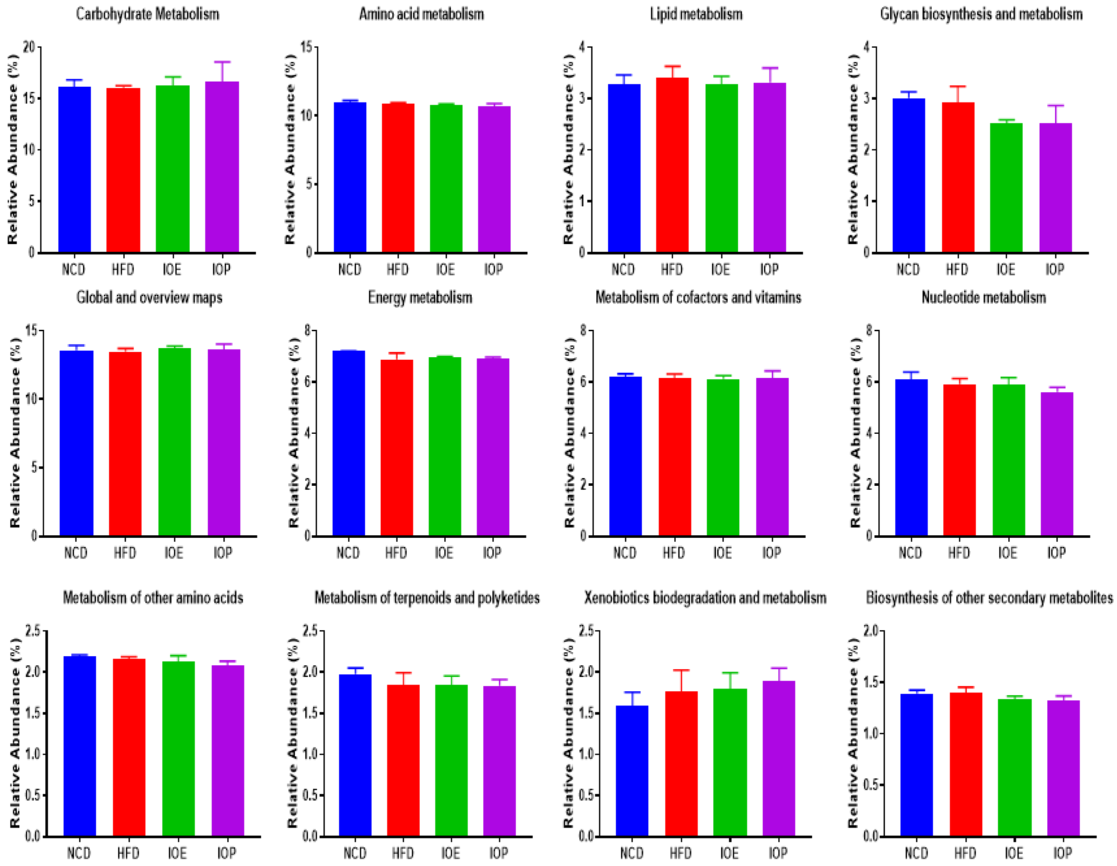

Figure S3. Statistical analysis of gut microbial predictive function (Class 2) of metabolism (Class 1) at week 14.

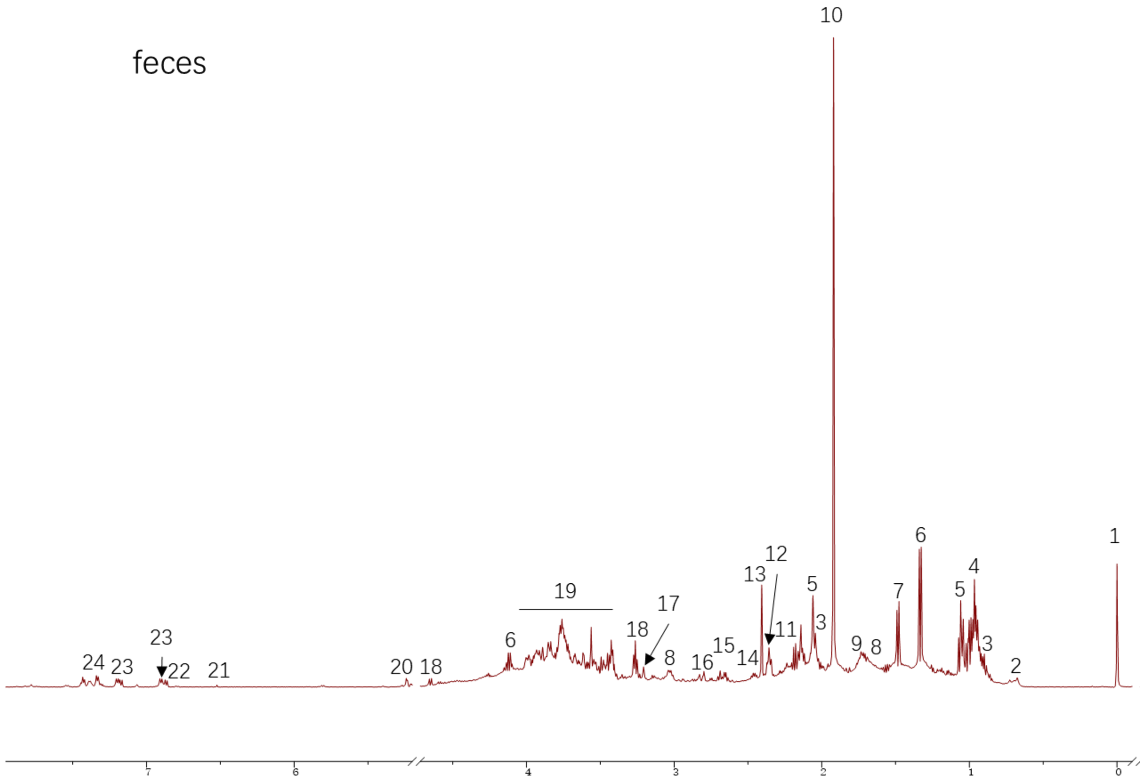

Figure S4. 600 MHz ^{1}H NMR spectra of mice urine. Keys : 1.TSP; 2.Bile acids; 3.Butyrate; 4.Bcaa; 5.Propionate; 6.Lactate ; 7.Alanine; 8.Lysine ; 9.5-aminovalerate ; 10.Acetate ; 11.Lipid; 12.Glutamate ; 13.Succinate; 14.Glutamine; 15.Citrate; 16.Aspartate; 17.Choline; 18.β-Glucose; 19.Glucose; 20.α-Glucose; 21.Fumarate; 22.Aminohippurate; 23.Tyrosine; 24.phenylacetyl glycine (PAG).

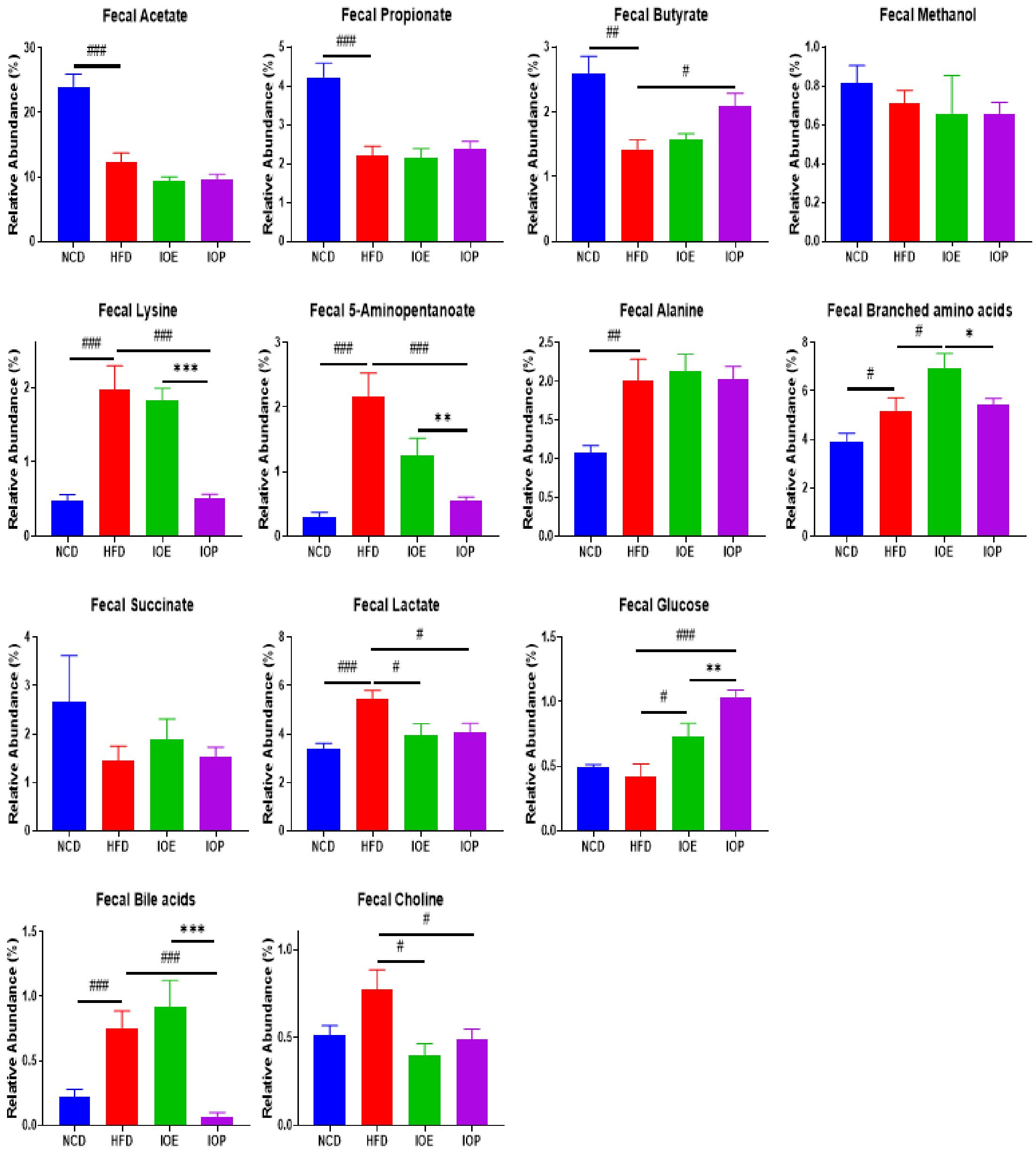

Figure S5. Main metabolites (VIP > 2) identified through ^{1}H NMR data of fecal samples at week 14. #: P<0.05 (vs. HFD); ##: P<0.01 (vs. HFD); ###: P<0.001 (vs. HFD); *: P<0.05 (IOE vs. IOP); **: P<0.01 (IOE vs. IOP); ***: P<0.001 (IOE vs. IOP).

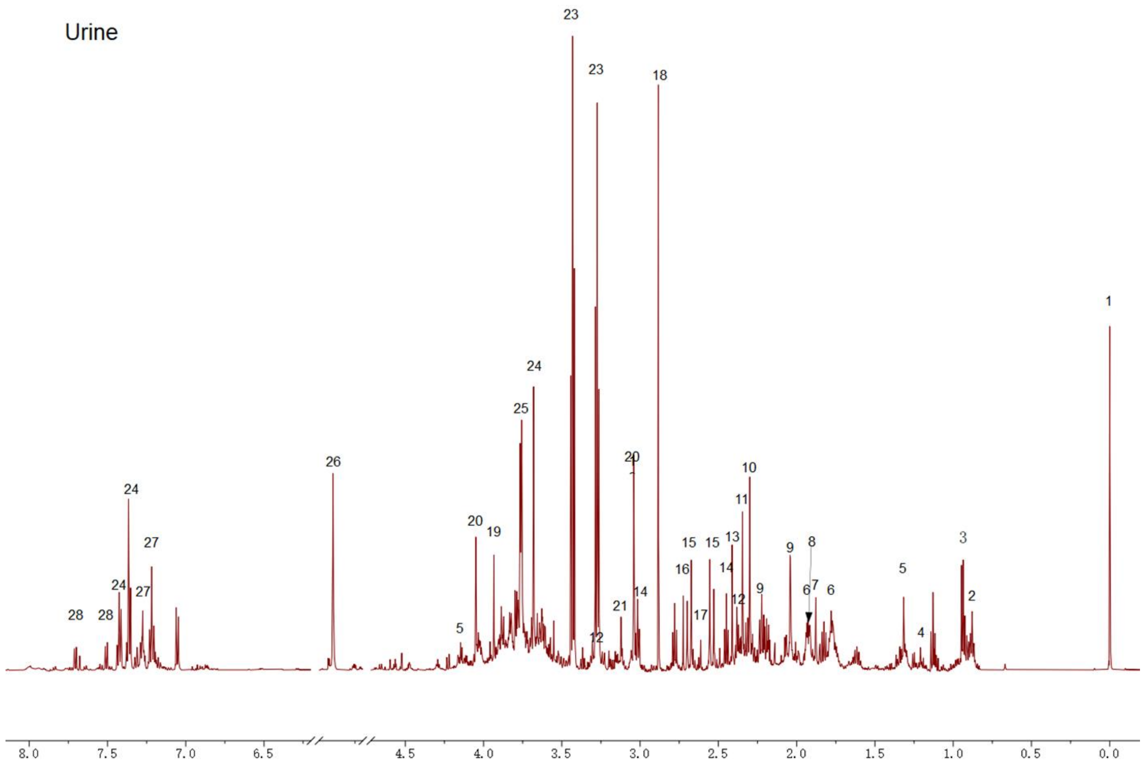

Figure S6. 600 MHz ^{1}H NMR spectra of mice urine. Keys: 1, TSP; 2, Butyrate; 3, Isoleucine; 4, 3-hydroxybutyrate; 5, Lactate; 6, Arginine; 7, Thymine; 8, Acetate; 9, N-acetylglutamate (NAG); 10, Acetoacetate; 11, Pyruvate; 12, 3-Ureidopropionate; 13, Succinate; 14, 2-Oxoglutarate; 15, Citrate; 16, Dimethylamine (TMA); 17, Methylamine; 18, Trimethylamine (TMA); 19, Creatine; 20, Creatinine; 21, Cis-aconitate; 22, Choline; 23, Taurine; 24, PhenylAcetyl Glycine (PAG); 25, Amino acid; 26, Allatoin; 27, 3-Indoxylsulfate; 28, N-methylnicotinamide (NMND).

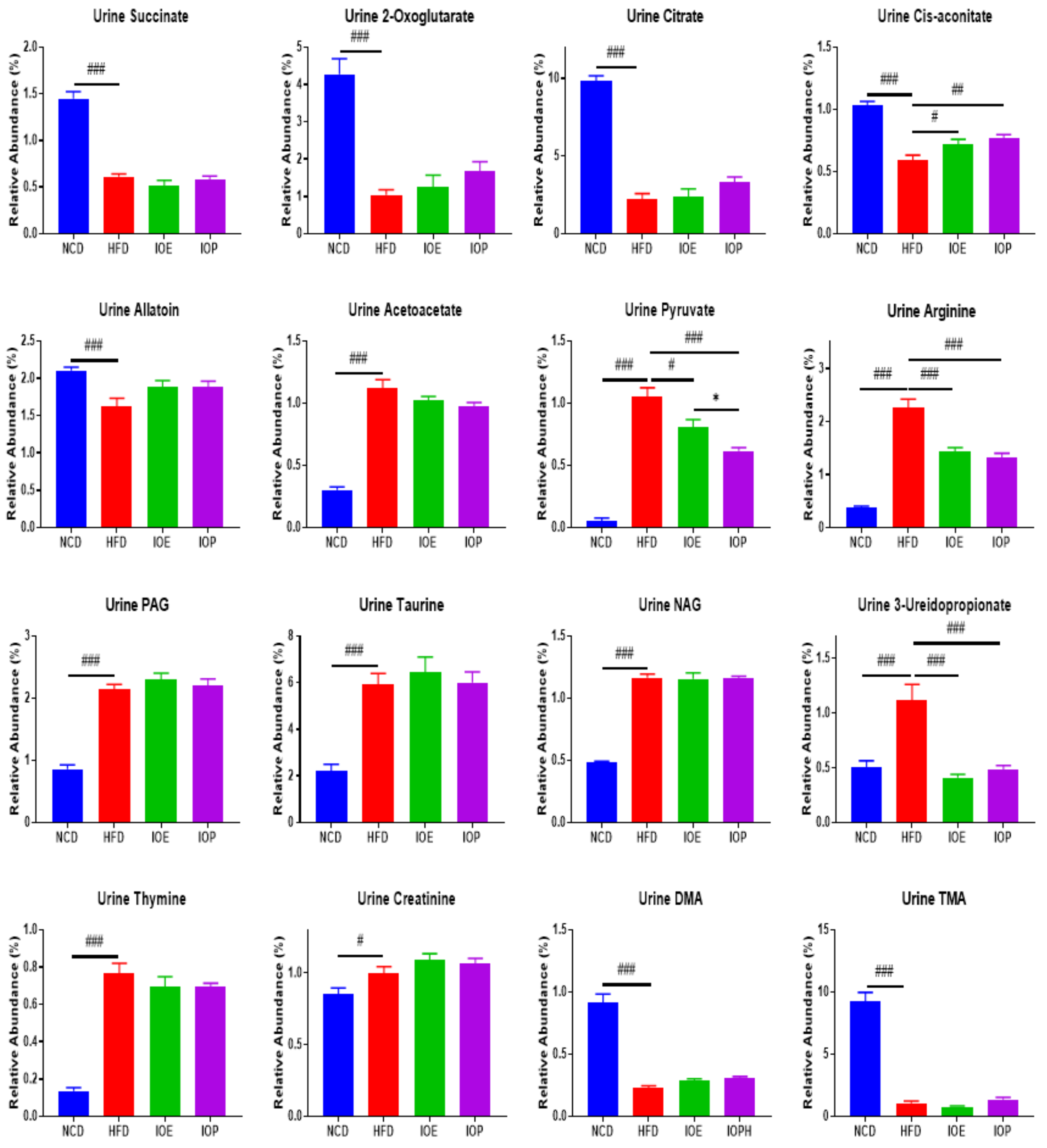

Figure S7. Main metabolites (VIP > 2) identified through ^{1}H NMR data of urine samples at week 14. #: P<0.05 (vs. HFD); ##: P<0.01 (vs. HFD); ###: P<0.001 (vs. HFD); *: P<0.05 (IOE vs. IOP); **: P<0.01 (IOE vs. IOP); ***: P<0.001 (IOE vs. IOP).

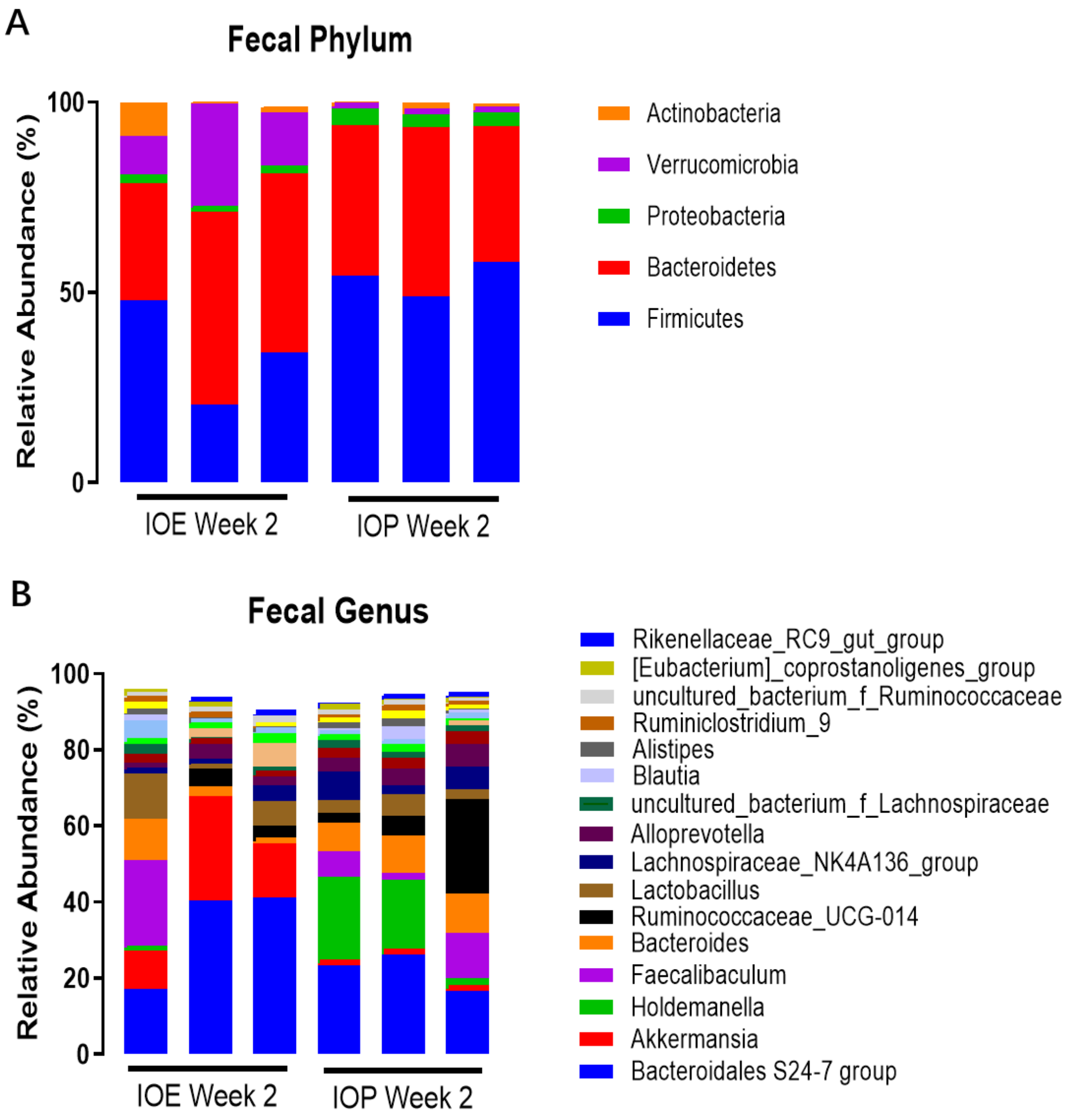

Figure S8. The composition of gut microbiota in the IOE group and the IOP group at week 2. (A) The composition of gut microbiota at phylum level; (B) The composition of gut microbiota at genus level
